# Polycystic Ovary Syndrome and Endometriosis as Reasons for Women’s Admission to Outpatient Specialist Care in Poland—A Retrospective Analysis

**DOI:** 10.3390/ijerph18041442

**Published:** 2021-02-04

**Authors:** Ewa Rzońca, Arkadiusz Kosowski, Agnieszka Bień, Joanna Gotlib, Arkadiusz Wejnarski, Marta Jarzębowska, Robert Gałązkowski, Patryk Rzońca

**Affiliations:** 1 Department of Education and Research in Health Sciences, Faculty of Health Sciences, Medical University of Warsaw, 02-091 Warsaw, Poland; joanna.gotlib@wum.edu.pl; 2 Department for Uniformed Services, Main Office of the National Health Fund in Warsaw, 02-390 Warsaw, Poland; ak.gabi@o2.pl; 3 Chair and Department of Development in Midwifery, Faculty of Health Sciences, Medical University of Lublin, 20-081 Lublin, Poland; agnesmbien@gmail.com; 4 Institute of Health Sciences, Faculty of Medical and Health Sciences, Siedlce University of Natural Sciences and Humanities, 08-110 Siedlce, Poland; arkadiusz.wejnarski@uph.edu.pl; 5 Polish Medical Air Rescue, 01-934 Warsaw, Poland; m.jarzebowska@lpr.com.pl; 6 Department of Emergency Medical Services, Faculty of Health Sciences, Medical University of Warsaw, 02-091 Warsaw, Poland; r.galazkowski@lpr.com.pl (R.G.); przonca@wum.edu.pl (P.R.)

**Keywords:** delivery of healthcare, polycystic ovary syndrome, appointments, endometriosis, outpatient clinics

## Abstract

This study aimed at presenting selected aspects of outpatient specialist care for women diagnosed with polycystic ovary syndrome (PCOS) or endometriosis. The study was carried out using a retrospective analysis of the services provided under Outpatient Specialist Care (AOS) for women, based on data from the National Health Fund (NFZ). The study included data on women with PCOS or endometriosis based on the International Statistical Classification of Diseases and Related Health Problems (ICD-10) in Poland from 2016 to 2018. The average age of women in the study group with PCOS was 25.31 (±7.02) years and, for those with endometriosis, 38.21 (±10.79). AOS patients with diagnosed PCOS most often made 2–3 visits (34.60%) to a specialist doctor, and those with diagnosed endometriosis most often made one visit (39.95%). Significant differences between patients with PCOS and endometriosis using AOS were found concerning the women’s age, the year, season, place of treatment, type of clinic, mode of admission, number of visits, and their place of residence or macroregion. With increasing age, women with PCOS made more visits to AOS, and women with endometriosis made fewer visits to the AOS specialist.

## 1. Introduction

Women’s health problems, especially gynaecological ones such as endometriosis and polycystic ovary syndrome (PCOS), have been the subject of numerous multifaceted studies [1,2,3,4,5,6,7,8,9,10,11,12]. Endometriosis is a disease whose pathogenesis has not been thoroughly explained [1,2,3,4]. This complex and heterogeneous disease is defined by the presence of endometrial-like tissue, consisting of stromal cells, glandular epithelium, or both, outside the uterus [5]. The disease occurs in about 10% of the female population, and is diagnosed in 20% to 90% of women with chronic pelvic pain or infertility. Additionally, endometriosis results in menstrual pain, pelvic pain, and dyspareunia. The disease is also strongly associated with infertility [1,2,3,4,5].

Polycystic ovary syndrome is also a disease whose aetiology is not fully understood. PCOS is also one of the causes of infertility in women and, similarly to endometriosis, occurs in an average of 10% of women. PCOS is characterized by several gynaecological, dermatological, and metabolic symptoms, including hirsutism, emotional disorders, and menstrual disorders [6,7,8,9]. It is diagnosed on the basis of two out of three criteria, namely, rare or no ovulation (menstrual disorders or the occurrence of a menstrual cycle without ovulation), the clinical and/or biochemical features of hyperandrogenism, and the presence of polycystic ovaries in ultrasound examination. These are the so-called Rotterdam criteria [7,8,9].

It should be noted that endometriosis and PCOS are chronic diseases with numerous symptoms that require appropriate and specialised health care because they affect the life and functioning of both the affected women and their relatives at different times of their lives [1,2,3,4,5,6,7,8,9]. The women who experience symptoms of these conditions seek specialist health care [1,3,9,10,11,12]. In Poland, the National Health Fund (NFZ) is a state organizational unit that deals with financing health services in the universal health care system. It has a legal character [13,14]. Primary Health Care (POZ) is the first and basic point of a patient’s contact with the health care system. POZ aims to provide comprehensive care for a woman’s health condition and undertakes many pro-health measures. The health care representatives who constitute this basic health care unit are the Primary Care Physician (GP), the Primary Care Nurse, and the Primary Care Midwife [15,16].

Besides these health care representatives, within the framework of Outpatient Specialist Care (AOS), it is possible to obtain specialist advice from a specialist doctor, an opinion on the patient’s state of health, as well as indications as to further proceedings on the basis of the diagnostic tests carried out. A referral is not required from a Primary Care Physician if the service recipient wants to use AOS services provided by a dentist, gynaecologist, obstetrician, oncologist, psychiatrist, or venereologist. When outpatient treatment is not sufficient, hospital treatment is necessary, and the patient may be referred to it. In contrast, no referral to the hospital is required for emergency admission [16].

Therefore, the problems of polycystic ovary syndrome and endometriosis, as well as the need for specialized health care, provided the rationale for why this research was conducted. It aimed to present selected aspects of Outpatient Specialist Care for women with diagnosed PCOS and endometriosis in Poland.

## 2. Materials and Methods

### 2.1. Study Design

The study was conducted using a retrospective analysis of services provided as part of the Outpatient Specialist Care for women based on data analysis from the documentation. The study included data on the women diagnosed with PCOS or endometriosis based on the International Statistical Classification of Diseases and Related Health Problems (ICD-10) in 2016–2018 in Poland. The study was conducted on the basis of data from the National Health Fund, which included the age of the patients, as well as the data characterizing the outpatient specialist care services, including the date of service and diagnosis of PCOS (E28.2) or endometriosis (N.80) based on the classification of ICD-10, mode of admission, type of clinic, and NFZ department. Based on the affiliation to a Branch of the National Health Fund, the examined women’s macroregion of residence was determined. Macroregions group the provinces in Poland into seven units: North (Pomorskie, Kujawsko-Pomorskie, and Warmińsko-Mazurskie Provinces), North-West (Zachodniopomorskie, Lubuskie, and Wielkopolskie Provinces), Central (Lódzkie and Świętokrzyskie Provinces), South-West (Dolnośląskie and Opolskie Provinces), South (Śląskie and Małopolskie Provinces), East (Podkarpackie, Podlaskie, and Lubelskie Provinces), and the Mazowieckie Province Macroregion [17].

In the analysed period of time, 19,954 women reported to AOS, of which 6044 were diagnosed with PCOS and 13,910 women were diagnosed with endometriosis. The data and consent of the National Health Fund were obtained for this research, and the retrospective nature of the study did not require the consent of the Bioethics Committee.

### 2.2. Statistical Analysis

The data obtained in the documentation analysis process were analysed statistically using Statistica (Tibco Software Inc., Palo Alto, CA, USA). The mean (M) and standard deviation (SD) were used to describe quantitative data, while the number (n) and percentage (%) were used to describe qualitative data.

The Kolmogorov–Smirnov and Lilliefors tests were used to verify the normal distribution of quantitative variables. The chi-squared test was used to assess statistically significant differences between qualitative variables. In contrast, the Mann–Whitney U non-parametric test was used to examine differences between two independent groups. The r-Pearson correlation was used to assess the relationship between selected variables, and the Guilford classification was used to assess the strength of the relationship [18]. To assess the chance of women receiving Outpatient Specialist Care for the analysed disease units, PCOS and endometriosis, to age and place of residence, the odds ratio (OR) and 95% confidence interval (95% CI) were used. Statistical significance was defined as *p* < 0.05.

## 3. Results

Table 1 presents the women’s age characteristics under examination and AOS care for patients with polycystic ovary syndrome (30.29%) and endometriosis (69.71%). The analysis shows that women between the ages of 18 and 29 with diagnosed PCOS were more likely to have received AOS care (66.41%); they visited a specialist in Outpatient Specialist Care mainly in spring (28.79%) and made 2–3 visits (34.60%) during the analysed period. On the other hand, the women with diagnosed endometriosis were more likely to be 30–39 years old (35.78%) and they were more likely to have received AOS care in winter (27.66%). Those respondents usually made one visit (39.95%) to a specialist in Outpatient Specialist Care. AOS care for women with PCOS and endometriosis was most often provided in 2016 (41.23% and 43.87%, respectively). Both the women diagnosed with polycystic ovary syndrome and endometriosis mainly used AOS in cities (97.57% and 94.16%) and most often visited obstetrics and gynaecology clinics (59.22% and 95%). They came to the doctor without a referral (57.93% and 95.09%), they lived (that is, belonged to the National Health Fund) in the South Macroregion (22.80% and 20.58%). Those women also benefited from the South Macroregion’s health care (23.81% and 20.47%).

**Figure 1:**
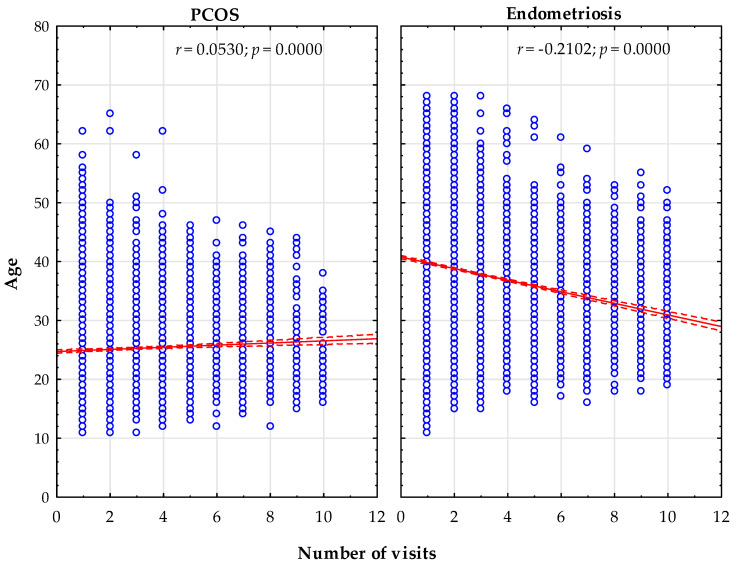
Correlation between the number of visits and the age of the women surveyed.

The statistical analysis showed a statistically significant, slight positive correlation (*r* = 0.0530) between the number of visits and the age of women treated for PCOS—the older the patient, the more visits to AOS. Additionally, a statistically significant, weak negative correlation (*r* = −0.2102) was found between the number of visits and the age of women treated for endometriosis in AOS—the older the patient, the fewer visits to a specialist (Figure 1).

Table 2 presents an analysis of the odds ratio of women with PCOS visiting a specialist in AOS in relation to the respondent’s age and their macroregion of residence. Under the age of 18, the ratio of women with PCOS to women with endometriosis was 5 times greater than the ratio of PCOS patients to endometriosis in the 18–29 age range, while in the remaining age groups, this ratio was lower. The ratio of women with PCOS living in the South, South-West, North-West, and East regions to women with endometriosis was greater than the ratio of women with PCOS to endometriosis patients living in the Mazowiecki region, and in the remaining groups, this ratio was smaller.

## 4. Discussion

Polycystic ovary syndrome and endometriosis are diseases characterized by numerous symptoms that have a negative impact on the life and functioning of women, so they seek specialized health care. The diseases have been the subject of numerous studies, and researchers are also looking for a relationship between these illnesses [1,2,3,4,5,6,7,8,9,10,11,12,19,20]. Therefore, in this paper our aim is to present selected aspects of Outpatient Specialist Care for women with diagnosed PCOS and endometriosis in Poland.

The literature on the health of women with endometriosis indicates that the women participating in the studies with this diagnosis were most often over 30 years of age, as was demonstrated in the research by, among others, De Graaff et al. (2013)—the average age of female respondents with endometriosis was 36.1 years [21]. Similar reports were presented by Simoens et al. (2012) [10]. Moradi et al. (2014), in their research on the impact of endometriosis on women’s lives, stated that the respondents were on average 31.1 years old [3]. Similar results from a study among Polish women were reported by Bień et al. (2020), where the women were on average 30.86 years old [22]. Our study results showed that the respondents with diagnosed endometriosis were on average 38.21 years old, while those with PCOS were on average 25.31 years old. The average age of women with PCOS in our study is similar to the average age of those reported by Moran et al. (2010), 22.41 years, and Lin et al. (2018), 28.2 years [6,23]. The difference between the studied groups may result, among other reasons, from the difficulty in diagnosing endometriosis, as the symptoms vary between women. Furthermore, the range of symptoms caused by the disease is extensive. In some women, the disease remains undiagnosed for a long time. Indeed, it may take up to 4–10 years from the first reported symptom to confirmation of the diagnosis [24].

The seasonality of health problems is an increasingly common aspect of research within the literature in the context of diseases, as pointed out by, among others, Hlimi (2015), who analysed the relationship between anaemia, pre-eclampsia, eclampsia, and seasonality [25]. Kim and Cheong (2019) investigated the issue of scabies and seasonality, Virág and Nyári (2018) undertook research on the annual and seasonal trends in cardiovascular mortality in Hungary, while Kyrgios et al. (2018) investigated the seasonality involving the month of the birth of children and adolescents with autoimmune thyroiditis [26,27,28]. The results of our research show that the women with PCOS came to the doctor within the framework of AOS, mainly in spring and, in the case of those with endometriosis, in winter.

PCOS and endometriosis are two frequently diagnosed separate clinical conditions; they are associated with extensive physical symptoms, have adverse psychological consequences, and are often associated with infertility [19,21]. Münster et al. (2018), in a study on the treatment of infertility in couples in Germany, showed that almost all women go to a gynaecologist before going to a fertility clinic for a consultation when they cannot have a child [29]. Our research results show that women diagnosed with PCOS and endometriosis under AOS mainly used the care of an obstetric, gynaecological clinic. They also reported to the doctor without a referral.

The Gibson-Helm et al. (2017) study on the experience of women who were diagnosed with polycystic ovary syndrome shows that nearly half of the women surveyed consulted one to two health care professionals before the diagnosis of the disease (PCOS) was made [9]. Additionally, Lin et al. (2018) made a study of women’s trust in physicians, which differentiated the women studied into those with and without PCOS. It showed that the surveyed women with polycystic ovary syndrome, in the context of visits involving general health in the last three years, mainly consulted primary care physicians. They were more likely to see a specialist doctor for PCOS problems [23]. Our research results found that women with polycystic ovary syndrome in the analysed period mainly made two to three visits. Our results also indicate that the older a woman with polycystic ovary syndrome is, the more visits she makes to the gynaecologist within AOS. The explanation for this correlation can be found in the fact that PCOS as a disease entity results in a number of problems, including reproductive health problems, dermatological problems, or metabolic abnormalities, including obesity, increased risk of cardiovascular disease, or diabetes. The treatment of the symptoms of this disease, and mitigation of potential long-term health complications, requires the use of health care involving physicians of various specialities [6,9,23,30].

With regard to the problem of endometriosis, Dancet et al. (2012), in a study on improving care for women with this disease, found that the time from the first symptoms to the diagnosis was, on average, 3.4 years. The number of consultations with the family doctor (GP) on the symptoms resulting from endometriosis before the patient was referred to a specialist was 7.3 visits. More than three-fifths of respondents were referred to specialists other than gynaecologists [11]. In turn, Apers et al. (2018) found that the average number of years from the onset of the first symptoms to the diagnosis of the disease was 4.2 years, and the number of consultations with GPs regarding the symptoms resulting from endometriosis before a referral to a specialist stood at 4.9 visits. Almost half of the women were consulted by more than one gynaecologist [31]. Bień et al. (2020), when analysing the quality of life and acceptance of the disease in Polish women with endometriosis, showed that most women consulted at least three doctors before a correct diagnosis was made [22]. However, our research results have shown that women with endometriosis most often make one visit to an AOS specialist. The older they become, the less often they visit an AOS specialist. Due to the non-characteristic symptoms occurring in endometriosis, the disease very often remains undiagnosed for a long time. Treatment is based on surgical procedures and/or hormone therapy. It aims to control the symptoms, and unfortunately, it does not always bring the expected long-term result [1,2,3,4,5,31,32,33]. Interesting in this context are the self-care measures for chronic diseases, which are an essential aspect in strengthening health, alleviating symptoms, and preventing the consequences of diseases, which can also apply to women with endometriosis [34,35]. These issues are reflected in the research presented by Armour et al. (2019) on self-management strategies for women with endometriosis in Australia. They have shown that self-management strategies and lifestyle modifications (e.g., use of warmth, rest, meditation, and breathing exercises) are a common and vital part of Australians’ self-care when they suffer from bothersome symptoms caused by the disease [33]. The search for professional medical care by women with endometriosis and, on the other hand, taking self-care measures may explain the results of our research, the correlation between the age of women with endometriosis, and the reporting to an AOS specialist [1,2,3,4,5,31,32,33,34,35].

Research problems concerning selected health aspects of Brazilian society presented in the studies of Azevedo e Silva et al. (2009), Tomazelli and Silva (2017), and Carreno et al. (2014) indicate that they are conditioned by numerous factors, including the nature of the macroregions and regions of Brazil, and their social and cultural context [36,37,38]. In turn, Wolf et al. (2018) conducted a study on the geographical prevalence of PCOS, which concluded that the analysed and existing data were not conclusive enough to determine whether there are significant differences in the prevalence of PCOS depending on different geographical locations and racial/ethnic group or origin [39]. However, our research found that the women with diagnosed PCOS and endometriosis mainly received outpatient specialist care in cities and received health care in the same macroregion they lived in—it was mainly the South Macroregion—and they also received health care there. Our research has also shown that the odds ratio of women with polycystic ovary syndrome reporting to AOS was higher for women under 18 and living in the South, South-West, North-West, and East Macroregions. It should be emphasized that both adolescence and PCOS are characterized by irregular menstrual cycles, lack of ovulation, and acne, which causes difficulties in making a diagnosis [40,41,42,43,44]. On the other hand, in the case of women with endometriosis, our research results have shown that the odds ratio in relation to reporting to a specialist under AOS is higher in women over 30 years of age and when living in the North and Central Macroregions. The literature on the subject increasingly emphasizes the issue of endometriosis in the perimenopausal period. It is a severe disease, which is increasingly occurring and has serious long-term health consequences, including a negative impact on the women’s quality of life and functioning [45,46,47,48,49].

The present study is the first retrospective analysis of health care in Poland in regard to Outpatient Specialist Care for women with polycystic ovary syndrome and endometriosis. The authors indicate that the study has some limitations. The analysis includes data made available only from the National Health Fund, without information concerning any sociodemographic factors, the health situation of the examined women, or the reasons for reporting to AOS, as well as further diagnostics, treatment, or care. However, these limitations do not affect the quality of the study. Further research should be conducted on the benefits provided to women under AOS, in the context of gynaecological problems, to understand these problems better and ensure the highest quality of care for women.

## 5. Conclusions

Significant differences between patients with PCOS and endometriosis receiving outpatient specialist care were found concerning the women’s age, the year, season, place of treatment, type of clinic, mode of admission, number of visits, and their area of residence (i.e., the macroregion). With increasing age, women with PCOS made more visits to AOS, and women with endometriosis made fewer visits to the AOS specialist.

The odds ratio of women with polycystic ovary syndrome reporting to AOS was higher for women under 18 and living in the South, South-West, North-West, and East Macroregions.

## Figures and Tables

**Table 1:** Characteristics and analysis of the age of women and Outpatient Specialist Care (AOS) care in patients with diagnosed endometriosis or polycystic ovary syndrome (PCOS).

Variables	PCOS 6044 (30.29)	Endometriosis 13,910 (69.71)	Total 19,954 (100.00)	*p*-Value
Age—n (%)				
Under 18 years old	584 (9.66)	76 (0.55)	660 (3.31)	0.0000
18–29 years old	4014 (66.41)	2891 (20.78)	6905 (34.60)	
30–39 years old	1197 (19.80)	4906 (35.78)	6103 (30.59)	
40–49 years old	227 (3.76)	4035 (29.01)	4262 (21.36)	
50–59 years old	16 (0.26)	1067 (7.67)	1083 (5.43)	
Over 60 years old	6 (0.10)	935 (6.72)	941 (4.72)	
Mean age (years old)—M (SD)	25.31 (7.02)	38.21 (10.79)	34.25 (11.45)	0.0000
Year—n (%)				
2016	2492 (41.23)	6103 (43.87)	8595 (43.07)	0.0000
2017	1727 (28.57)	4207 (30.24)	5934 (29.74)	
2018	1825 (30.20)	3600 (25.88)	5425 (27.19)	
Season of the year—n (%)				
Spring	1740 (28.79)	3781 (27.18)	5521 (27.67)	0.0014
Summer	1388 (22.96)	3037 (21.83)	4425 (22.18)	
Autumn	1389 (22.98)	3245 (23.33)	4634 (23.22)	
Winter	1527 (25.26)	3847 (27.66)	5374 (26.93)	
Place of treatment—n (%)				
City	5897 (97.57)	13,098 (94.16)	18,995 (95.19)	0.0000
Village	147 (2.43)	812 (5.84)	959 (4.81)	
Type of clinic—n (%)				
Obstetrics and gynaecology clinic	3579 (59.22)	13,214 (95.00)	16,793 (84.16)	0.0000
Endocrinology clinic	2445 (40.45)	61 (0.44)	2506 (12.56)	
Another clinic	20 (0.33)	635 (4.57)	655 (3.28)	
Mode of admission—n (%)				
No referral	3501 (57.93)	13,227 (95.09)	16,728 (83.83)	0.0000
With referral	2543 (42.07)	683 (4.91)	3226 (16.17)	
Number of visits—n (%)				
1 visit	2036 (33.69)	5557 (39.95)	7593 (38.05)	0.0000
2–3 visits	2091 (34.60)	4172 (29.99)	6263 (31.39)	
4–5 visits	960 (15.88)	1824 (13.11)	2784 (13.95)	
6 or more visits	957 (15.83)	2357 (16.94)	3314 (16.61)	
Number of visits—M (SD)	3.04 (2.43)	2.96 (2.61)	2.99 (2.55)	0.0000
Macroregion of residence—n (%)				
South Macroregion	1378 (22.80)	2862 (20.58)	4240 (21.25)	0.0000
South-West Macroregion	856 (14.16)	902 (6.48)	1758 (8.81)	
North-West Macroregion	1034 (17.11)	1940 (13.95)	2974 (14.90)	
North Macroregion	545 (9.02)	2379 (17.10)	2924 (14.65)	
Central Macroregion	566 (9.36)	1810 (13.01)	2376 (11.91)	
East Macroregion	1064 (17.60)	2394 (17.21)	3458 (17.33)	
Mazowieckie Province Macroregion	601 (9.94)	1623 (11.67)	2224 (11.15)	
Macroregion of treatment—n (%)				
South Macroregion	1439 (23.81)	2848 (20.47)	4287 (21.48)	0.0000
South-West Macroregion	853 (14.11)	897 (6.45)	1750 (8.77)	
North-West Macroregion	1084 (17.94)	1927 (13.85)	3011 (15.09)	
North Macroregion	480 (7.94)	2377 (17.09)	2857 (14.32)	
Central Macroregion	519 (8.59)	1809 (13.01)	2328 (11.67)	
East Macroregion	1045 (17.29)	2389 (17.17)	3434 (17.21)	
Mazowieckie Province Macroregion	624 (10.32)	1663 (11.96)	2287 (11.46)	
Treatment in the macroregion of residence—n (%)				
Yes	5476 (90.60)	13,277 (95.45)	18,753 (93.98)	0.0000
No	568 (9.40)	633 (4.55)	1201 (6.02)	

PCOS—polycystic ovary syndrome.

**Table 2:** Visits of women with PCOS to AOS in relation to age and macroregion of residence.

Variables	PCOS		
	OR	95% CI	*p*-Value
Age (reference group: 18–29 years old)			
Under 18 years old	5.53	4.34–7.06	0.0000
30–39 years old	0.17	0.16–0.19	0.0000
40–49 years old	0.04	0.04–0.05	0.0000
50–59 years old	0.01	0.01–0.02	0.0000
Over 60 years old	0.01	0.00–0.01	0.0000
Macroregion of residence (reference group: Mazowieckie Province Macroregion)			
South Macroregion	1.30	1.16–1.46	0.0000
South-West Macroregion	2.56	2.25–2.93	0.0000
North-West Macroregion	1.44	1.28–1.62	0.0000
North Macroregion	0.62	0.54–0.71	0.0000
Central Macroregion	0.84	0.74–0.97	0.0127
East Macroregion	1.20	1.07–1.35	0.0025

## Data Availability

Data not available due to restrictions according to the NFZ (National Health Fund).
